# Metronidazole-mediated gut anaerobe remodeling is associated with transplant rejection

**DOI:** 10.3389/fcimb.2026.1874387

**Published:** 2026-08-18

**Authors:** Yanzhuo Liu, Yang Tian, Chunlan Pu, Yan Yang, Xinhao Peng, Hai Zhu, Jia Fan, Rongke Zhang, Hao Yuan, Jun Zhang, Jianyu Liu, Gaoping Zhao

**Affiliations:** 1 Department of Oncology, The Affiliated Hospital of Southwest Jiaotong University, The Third People’s Hospital of Chengdu, Chengdu, Sichuan, China; 2 Medical Research Center, The Affiliated Hospital of Southwest Jiaotong University, The Third People’s Hospital of Chengdu, Chengdu, Sichuan, China; 3 The Seventh People’s Hospital of Chengdu Affiliated Cancer Hospital of Chengdu Medical College, Chengdu, Sichuan, China; 4 Department of Gastrointestinal Surgery, Sichuan Provincial People’s Hospital, University of Electronic Science and Technology of China, Chengdu, Sichuan, China

**Keywords:** anaerobic bacteria, glycosylation, metronidazole, Th17 response, transplant rejection

## Abstract

**Background:**

The gut microbiome has emerged as a potential modulator of transplant rejection. However, the relevance of anaerobe-associated gut microbial communities to transplant rejection responses remains unclear.

**Methods:**

In a murine islet transplantation model, we assessed the effects of metronidazole pretreatment on allograft survival and function. 16S rRNA sequencing was performed to characterize treatment-associated changes in gut microbial composition. Separately, an exploratory two-sample MR analysis using human GWAS data was performed to prioritize gut microbial taxa associated with a composite outcome of transplant failure or rejection. Biological annotation was subsequently used to prioritize candidate host pathways. Immunohistochemistry and flow cytometry were used to evaluate post-transplant immune responses after metronidazole pretreatment.

**Results:**

Metronidazole treatment remodeled gut microbiota composition and significantly delayed islet allograft rejection. MR analyses identified several anaerobe-associated gut microbial taxa potentially relevant to transplant rejection risk, including the family *Defluviitaleaceae* and the genera *Intestinibacter*, *Bilophila*, *Ruminococcus* and *Eubacterium fissicatena*. Integrative biological annotation highlighted glycosylation-related pathways and prioritized ST3GAL4 for subsequent expression assessment in the murine transplant model. Furthermore, metronidazole treatment reduced CD4^+^ T-cell infiltration around the allograft and Th17-related inflammatory responses.

**Conclusion:**

Altogether, our findings document gut microbial remodeling and delayed allograft rejection following metronidazole pretreatment. These findings are hypothesis-generating and warrant further mechanistic investigation.

## 1 Introduction

Solid organ transplantation stands as the best therapeutic option for patients with end-stage organ failure; however, graft rejection continues to limit long-term transplant outcomes. Donor–recipient genetic mismatch remains a central determinant of graft rejection, as donor-derived allo-antigens are recognized by alloreactive recipient T cells and trigger the activation of protective immune responses (Lei et al., 2016). In addition to genetic disparities, environmental factors (such as pollutants, microbiota, high-salt and high-fat diets) can modulate alloimmune responses and accelerate transplant rejection (Safa et al., 2015; Molinero et al., 2016). Accordingly, elucidating the mechanisms by which environmental cues shape alloimmune responses may inform the development of novel therapeutic strategies to prevent or ameliorate transplant rejection.

Previous studies have linked infection to an increased incidence of acute rejection in kidney and lung transplant patients (Abbott et al., 2004; Husain et al., 2007). Given that commensal microbes and pathogens share overlapping molecular features capable of shaping host immune responses, the gut microbiota has emerged as a potential contributor to allograft dysfunction. Several studies reported that organs with extensive microbial colonization, such as the intestine and lung, tend to exhibit poorer transplant outcomes than relatively sterile organs, such as the heart and kidney (Alegre et al., 2014; Geva-Zatorsky et al., 2017). Evidence from germ-free or antibiotic-mediated microbiota-depletion models has shown prolonged skin-graft survival, whereas reconstitution of germ-free mice with fecal microbiota from conventional colonized donors accelerates graft rejection (Belkaid and Hand, 2014; Lei et al., 2016). These findings indicate that the microbiota can influence graft outcome in transplantation.

Anaerobic bacteria exist as part of the normal flora in the human intestinal tract, oral cavity and urogenital tract, and can cause infectious diseases as a result of impairment to the microenvironment (Maier et al., 2014). It has been suggested that anaerobic infection has been clinically recognized as a potential component of early post-transplant infectious episodes in liver transplant recipients (Philpott-Howard et al., 2003). Notably, enrichment of oral-derived anaerobes is associated with early graft dysfunction and rejection-related processes after lung transplantation (Snyder and Kitsios, 2024). These results indicated that anaerobe-associated microbial communities may represent an important link between microbial dysbiosis and transplant outcomes. Metronidazole is a nitroimidazole antibiotic primarily used to treat or prevent systemic or local infections caused by anaerobic bacteria (Löfmark et al., 2010; Chen et al., 2025). Clinical evidence suggests that metronidazole may influence transplant outcomes by modulating anaerobic bacterial communities (Philpott-Howard et al., 2003). However, whether pre-transplant perturbation of the anaerobic microbiota through metronidazole treatment is associated with transplant rejection remains poorly understood.

Our group developed multiple allograft transplantation models, including islet, heart, skin, and kidney models, and these models have been used to explore the mechanisms of immune tolerance and rejection in transplant medicine (Zhao et al., 2011; Liu et al., 2025). Here, we used a murine islet transplantation model to examine whether metronidazole-mediated gut microbiota remodeling is associated with allograft outcome and post-transplant immune responses. Our results showed that metronidazole treatment prolonged islet allograft survival and broadly reshaped gut microbial composition. In parallel, two-sample Mendelian randomization identified gut microbial taxa associated with rejection risk, while integrative annotation highlighted glycosylation-related host pathways, including ST3GAL4, ST3GAL6, and B4GALT4. In the transplant setting, metronidazole-mediated microbiota remodeling was accompanied by reduced graft ST3GAL4 expression and diminished CD4^+^Th17-associated immune responses. These findings provide complementary experimental and human genetic observations for exploratory candidate prioritization.

## 2 Materials and methods

### 2.1 Animals

Wild-type mice (WT) on a C57BL/6J background were purchased from Cyagen Biosciences (Suzhou, China). BALB/c mice, which served as islet allograft donors, were also obtained from the same vendor. All mice used in the experiments were 6–10 weeks old and were housed under controlled environmental conditions with a 12-hour light/dark cycle. Mice were maintained under specific pathogen-free conditions with free access to food and water. Because mice are coprophagic and co-housing can facilitate microbial exchange among cage mates, recipient mice were housed individually throughout the experimental period. Each treatment group comprised five recipient mice, with each mouse housed individually in a separate ventilated cage and provided with an independent water bottle (**Supplementary Table 1**).

For microbiome analysis, fecal samples were collected individually from each mouse at a fixed time window and were not pooled. Samples were freshly collected and immediately processed or stored at −80 °C until DNA extraction.

All animal experiments were approved by the Institutional Animal Care and Use Committee of the Affiliated Hospital of Southwest Jiaotong University and were performed in accordance with relevant national regulations and institutional guidelines.

### 2.2 Antibiotic treatment and islet transplantation

WT mice were treated with metronidazole (1 g/L, Macklin, Shanghai, China) added to the drinking water for two consecutive weeks before transplantation (Fujisaka et al., 2016). After two weeks of pretreatment, WT mice received a single intraperitoneal injection of streptozotocin (STZ, 200 mg/kg; Sigma, St. Louis, MO, USA) to induce diabetes. Transplant recipients were considered diabetic when their blood glucose levels exceeded 300 mg/dL (>18.8 mmol/L) for at least two consecutive days. Mice meeting this criterion were then used for islet transplantation.

Pancreatic islets from 2–3 donor BALB/c mice were isolated using 1.5 mg/mL cold collagenase followed by Ficoll density gradient purification (densities: 1.11, 1.096, 1.066). Freshly isolated islets were stained with dithizone (DTZ) and inspected under a stereomicroscope. Approximately 500 fresh islets were transplanted under the kidney capsules of each diabetic recipient. Euglycemia was defined as a non-fasting blood glucose level below 200 mg/dL (< 11.1 mmol/L). Graft rejection was defined as recurrent hyperglycemia, with blood glucose levels exceeding 200 mg/dL (>11.1 mmol/L) in at least two consecutive measurements (Yuan et al., 2025).

### 2.3 Immunohistochemistry

The engrafted kidneys were harvested by nephrectomy from WT mice with or without metronidazole treatment. The engrafted kidney was fixed in 10% neutral buffered formalin overnight. Immunostaining procedures were performed using a standard approach (Guo et al., 2022). Briefly, 4-6 μm paraffin-embedded tissue sections were deparaffinized and washed three times with PBS. Sections were blocked with blocking buffer (Thermo Fisher Scientific, USA). Then, primary antibodies against anti-CD4 and anti-CD8 (1:200, Abcam, USA) were applied to sections and incubated in a humidified chamber at room temperature for 2 hours (h). After washing, sections were incubated with biotinylated secondary antibodies (Cell Signaling Technology, USA) at room temperature for 1 h, followed by streptavidin–HRP incubation (Cell Signaling Technology, USA) for 30 min in the dark. These sections were dehydrated with 95% ethanol and 100% ethanol. A DAB substrate solution (Cell Signaling Technology, USA) was applied for chromogenic development according to the manufacturer’s instructions. Sections were then dehydrated through graded ethanol, cleared in xylene, and covers lipped. CD4^+^- and CD8^+^-positive cells were counted in randomly selected fields using Image-Pro Plus, and the mean count across fields was calculated for each graft. Each recipient mouse was treated as the biological unit for statistical analysis.

### 2.4 16S rRNA sequencing and analysis

Fecal samples were collected on day 3 after transplantation. Amplification and DNA sequencing of the 16S rRNA gene were performed by Shanghai Majorbio Bio-Pharm Technology Co., Ltd (Shanghai, China). For beta-diversity analysis, principal component analysis (PCA) was performed on the normalized OTU abundance matrix to visualize community-level differences between groups using a Euclidean-based linear ordination. In addition, Bray–Curtis distances were calculated from OTU-level abundance profiles and used for principal coordinate analysis (PCoA). Between-group differences in microbial community structure were assessed using PERMANOVA. A detailed description of the 16S rRNA sequencing and downstream bioinformatic analyses is provided in the online supplement, SDC (http://links.lww.com/TP/B549).

### 2.5 Quantitative real-time RT-PCR

Total RNA was extracted using TRIzol reagent (Thermo Fisher Scientific, USA) and reverse-transcribed into cDNA using a First-Strand cDNA Synthesis Kit (Vazyme, Nanjing, China) according to the manufacturer’s instructions. Quantitative PCR was performed using 2× SYBR Green qPCR Master Mix (Vazyme, Nanjing, China) on an ABI 7500 Real-Time PCR System (Applied Biosystems, USA). Target mRNA expression levels were calculated using the 2^-ΔΔCt^ method, with GAPDH as the endogenous control. Primer sequences used for qRT-PCR are listed in **Table 1**.

**Table 1:** Primer sequences used for qRT-PCR.

Gene	Forward primer	Reverse sequence
ST3GAL4	5’-TGCCTCCAACAAGAAGCAGACC-3’	5’-AGCATCCGCTTGATGGCGATAG -3’
B4GALT4	5’-TGCAGAGGCAACAGCTGGACTA-3’	5’-CGTCGTGGAATACGAAGCAGTC-3’
IFN-γ	5’-CAGCAACAGCAAGGCGAAAAAGG-3’	5’-TTTCCGCTTCCTGAGGCTGGAT-3’
IL-4	5’-ATCATCGGCATTTTGAACGAGGTC-3’	5’-ACCTTGGAAGCCCTACAGACGA-3’
IL-22	5’-GCTTGAGGTGTCCAACTTCCAG-3’	5’-ACTCCTCGGAACAGTTTCTCCC-3’
IL-17a	5’-CAGACTACCTCAACCGTTCCAC-3’	5’-TCCAGCTTTCCCTCCGCATTGA-3’
IL-1β	5’-TGGACCTTCCAGGATGAGGACA-3’	5’-GTTCATCTCGGAGCCTGTAGTG-3’

### 2.6 Flow cytometry

The single-cells were isolated from the spleen and mesenteric lymph nodes by filtration through a 70 μm nylon mesh and were prepared into suspensions. Erythrocytes were lysed in cold red blood cell (RBC) lysis buffer (BioLegend, USA, San Diego) and the collected cells were washed with PBS. The cells were counted using a hemocytometer.

Nonspecific binding was blocked with an anti-CD16/CD32 antibody. For surface marker analysis, the cells described above were stained with fluorophore-conjugated monoclonal antibodies against specific cell surface markers, including anti-CD3 (BD Biosciences), anti-CD4 (BD Biosciences) antibodies which were purchased from BD (USA, New Jersey, USA). For detection of the intracellular cytokines IFN-γ and interleukin (IL)-17A, cells were suspended in buffer containing 0.1% azide and 2% fetal bovine serum; stimulated with the components of a Stimulation Cocktail Kit containing phorbol 12-myristate 13-acetate (PMA), ionomycin, and a protein transport inhibitor (1:500, eBioscience, San Diego, CA, USA) at 37 °C for 5 hours, followed by fixation and permeabilization using a Fixation/Permeabilization Kit according to the manufacturer’s instructions. The samples were then intracellular staining for IFN-γ (BD Biosciences) and IL-17A (BD Biosciences) following the antibody manufacturer’s protocol (eBioscience, California, USA). The samples were injected into a FACSVerse flow cytometer (Becton Dickinson) and the data were analyzed with FlowJo software (BD Biosciences San Jose, California, USA).

### 2.7 Study design, data sources and instrumental variable selection

Mendelian randomization (MR) is a data analysis method primarily utilized in epidemiological etiology inference. According to the Mendelian independent distribution law, alleles are randomly assigned to progeny gametes during gamete formation. This characteristic ensures that the associations between genes and diseases remain unaffected by common confounding factors, and the causal timing is reasonable, resulting in effect estimates that closely resemble real-world situations. To the best of our knowledge, no previous study has investigated the association between gut microbiota and graft function after transplantation using Mendelian randomization analysis. Here, we employed the two-sample MR approach to evaluate genetically predicted associations between gut microbiota and graft dysfunction following organ and tissue transplantation. The schematic representation of the study design is presented in **Supplementary Figure 1**, showing the two-sample MR analysis of 198 gut microbial taxa and transplant outcomes. This examination was conducted in accordance with the guidelines provided by the STROBE-MR checklist (**Supplementary Table 2**). Sensitivity analyses and filtering of single nucleotide polymorphisms (SNPs) were conducted in accordance with the recommended protocols (Sanderson et al., 2022). The genetic data for gut microbiome were obtained from the latest GWAS summary statistics generated by the MIBioGen consortium, which curated and analyzed genome-wide genotypes data and 16S rRNA fecal microbiome profiles from 14,306 individuals across 24 cohorts. This dataset included 198 bacterial taxa across multiple taxonomic levels (119 genera, 34 families, 20 orders, 16 classes, and 9 phyla). We selected GWAS summary statistics for failure and rejection of transplanted organs and tissues from the FinnGen cohort (ST19_FAILU_REJEC_TRANSPLANTED_ORGANS_TISSU; 124 cases and 199,271 controls), which is defined using ICD-10 code T86 in national hospital discharge and cause-of-death registries (https://gwas.mrcieu.ac.uk/). All datasets analyzed in this study were derived from individuals of European ancestry to reduce potential bias from population stratification. Because the FinnGen T86 phenotype is based on registry data, detailed information regarding transplanted organ types, immunosuppressive regimens, and histopathological rejection subtypes was unavailable. The relatively limited number of rejection cases may have reduced statistical power and affected the precision of effect estimates, particularly for microbial taxa with modest genetically predicted effects. Therefore, these findings should be interpreted as exploratory evidence of broad transplant-related genetic associations rather than evidence specific to islet transplantation or any individual transplanted organ. Detailed descriptions of the datasets and their characteristics are provided in **Supplementary Table 3**.

The following selection criteria were used to choose the IVs: (1) single nucleotide polymorphisms (SNPs) associated with each microbial taxon at the locus-wide significance threshold (P < 1.0×10^-5^) were selected as potential IVs (Sanna et al., 2019; Yang et al., 2024); (2) 1000 Genomes project European samples data were used as the reference panel to calculate the linkage disequilibrium (LD) between the SNPs, and among those SNPs exhibiting an LD threshold r^2^ < 0.001 (clumping window size=10,000 kb), only the SNPs with the lowest P-values were retained; (3) when palindromic SNPs existed, the forward strand alleles were inferred using allele frequency information. To minimize the impact of weak instrument bias on causal inference, the F-statistic values were all above 10. The Steiger filter was applied to remove SNPs with a larger R^2^ in allograft transplant rejection than gut microbial taxa. The information on the SNP selection process is provided (**Supplementary Table 4**). These steps were used to improve the independence, relevance, and validity of the genetic instruments, satisfying the core assumptions of Mendelian randomization.

### 2.8 Biological annotation

For exploratory biological annotation, taxa prioritized by the human MR analysis and also detectable in the murine 16S dataset were retained, irrespective of whether they met the differential-abundance threshold in mice. SNPs associated with these taxa were extracted from the MiBioGen microbiome GWAS summary statistics using the same locus-wide threshold applied in the MR analysis (P < 1 × 10^-5^). These SNPs were then mapped to positional candidate genes using the FUMA platform (https://fuma.ctglab.nl/). The resulting genes were subsequently evaluated using tissue-expression, protein–protein interaction, and functional enrichment analyses.

Tissue-specific expression enrichment was evaluated using Genotype Tissue Expression (GTEx v8)-based hypergeometric tests implemented in FUMA (The GTEx Consortium, 2020). Protein–protein interaction (PPI) networks were then constructed using STRING v11.5 (Szklarczyk et al., 2021). The largest connected component of the PPI network was extracted to avoid fragmented subnetworks, and visualized in Cytoscape (Shannon et al., 2003). Hub genes were defined as the top 12 nodes by the Maximal Clique Centrality (MCC) method in cytoHubba, as highly connected genes contribute to network stability (Chin et al., 2014). Bulk tissue expression profiles of the hub genes were evaluated using GTEx v8 portal (Yu et al., 2019). Pleiotropic associations were further evaluated across 4,756 complex traits from 28 domains using phenome-wide association studies (PheWAS) in GWAS ATLAS (Watanabe et al., 2017). Finally, positional candidate genes mapped from IVs associated with taxa prioritized by the MR analysis and detected in the murine 16S dataset were annotated using the GWAS Catalog implemented in FUMA to explore previously reported phenotype associations.

### 2.9 Statistical analysis

Data are presented as mean ± SD unless otherwise specified. Statistical significance between two groups was determined using an unpaired two-tailed Student’s *t*-test unless otherwise specified. Graft survival was analyzed using the Kaplan–Meier method and compared using the log-rank test. For glucose tolerance analysis, the area under the curve (AUC) was calculated from the glucose tolerance test. Differences in beta-diversity between groups were evaluated using PERMANOVA based on the Bray–Curtis distance matrix. Data analysis was performed using GraphPad Prism 6 (GraphPad Software, USA). A *P* value < 0.05 was considered statistically significant (*P < 0.05, **P < 0.01, ***P < 0.001, and ****P < 0.0001). *P* > 0.05 was considered not significant (n.s.).

The primary MR estimates in the two-sample MR analyses were derived using the random-effects inverse-variance weighted method. To assess the consistency of the results and the potential influence of invalid or pleiotropic instruments, we applied complementary MR approaches (Bowden et al., 2015). Specifically, MR-Egger regression evaluates directional pleiotropy under the Instrument Strength Independent of Direct Effect assumption; the weighted median estimator provides consistent estimates when at least 50% of the weight comes from valid instruments; and the weighted mode estimator identifies the most common effect among a valid subset of instruments (Bowden et al., 2016). These methods were used to examine the consistency of the MR findings across different model assumptions. Heterogeneity across SNP instruments was assessed using Cochran’s Q statistic, with P < 0.05 indicating heterogeneity. All MR analyses were performed in R version 3.6.0 using the Two-Sample MR package (RRID: SCR_019010). The use of complementary estimators and sensitivity analyses ensured the consistency and stability of the MR estimates. Directional horizontal pleiotropy was assessed using the MR-Egger intercept test. Leave-one-out analyses were performed to determine whether the MR estimates were disproportionately influenced by individual SNP instruments.

## 3 Results

### 3.1 Metronidazole pretreatment prolongs islet allograft survival

**Figure 1:**
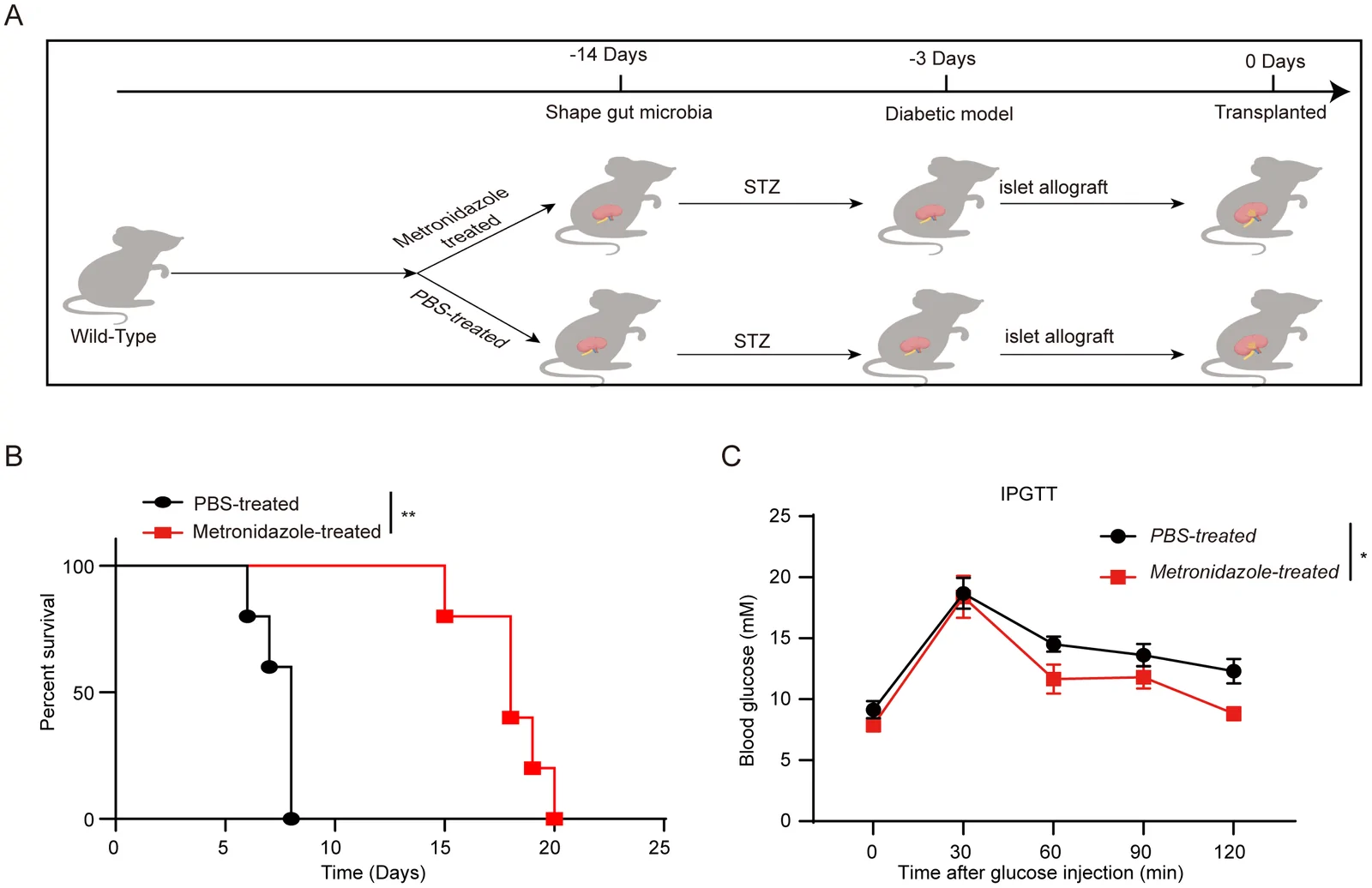
Metronidazole pretreatment delays islet allograft rejection and improves post-transplant graft function. **(A)** Schematic diagram of experimental design. Metronidazole or PBS was administered for 14 consecutive days to modulate gut microbiota composition. Diabetes was induced by streptozotocin (STZ) injection three days prior to islet allograft transplantation. Islet transplantation was then performed, and graft survival and function were subsequently monitored. **(B)** Kaplan–Meier survival curves showing islet allograft survival in metronidazole-treated (n=5) and PBS-treated (n=5) groups after transplantation. Graft rejection was defined as recurrent hyperglycemia, with blood glucose levels >200 mg/dL (>11.1 mmol/L) for at least two consecutive readings. **(C)** Intraperitoneal glucose tolerance tests (IPGTTs) were performed in metronidazole-treated and PBS-treated recipient mice after islet transplantation, and results are presented as the mean ± SD. AUC values were compared using an unpaired two-tailed Student’s t-test. *p < 0.05, **p < 0.01.

To determine whether metronidazole-mediated gut microbiota remodeling was associated with allograft outcomes *in vivo*, diabetic C57BL/6J mice were pretreated with metronidazole for 14 consecutive days before transplantation (Metronidazole-treated), while control mice received PBS (PBS-treated), and graft survival was subsequently monitored (**Figure 1A**). Blood glucose monitoring was used to assess graft function and determine the time to rejection post-transplantation. Graft rejection was defined as recurrent hyperglycemia, with blood glucose levels greater than 200 mg/dL (>11.1 mmol/L) for at least two consecutive readings. PBS-treated recipients rapidly developed hyperglycemia, indicating loss of islet allograft function, with a mean graft survival time of 7.4 ± 0.9 days. In contrast, metronidazole-treated recipients also eventually reached the rejection criterion, but at later time points, resulting in prolonged islet allograft survival with a mean graft survival time of 18.0 ± 1.9 days (**Figure 1B**). Intraperitoneal glucose tolerance tests (IPGTTs) demonstrated improved glucose tolerance in metronidazole-treated mice compared with PBS-treated controls (**Figure 1C**). These data indicate that metronidazole pretreatment was associated with prolonged graft survival and improved post-transplant glycemic control.

### 3.2 Metronidazole reshapes gut microbiota composition and diversity

**Figure 2:**
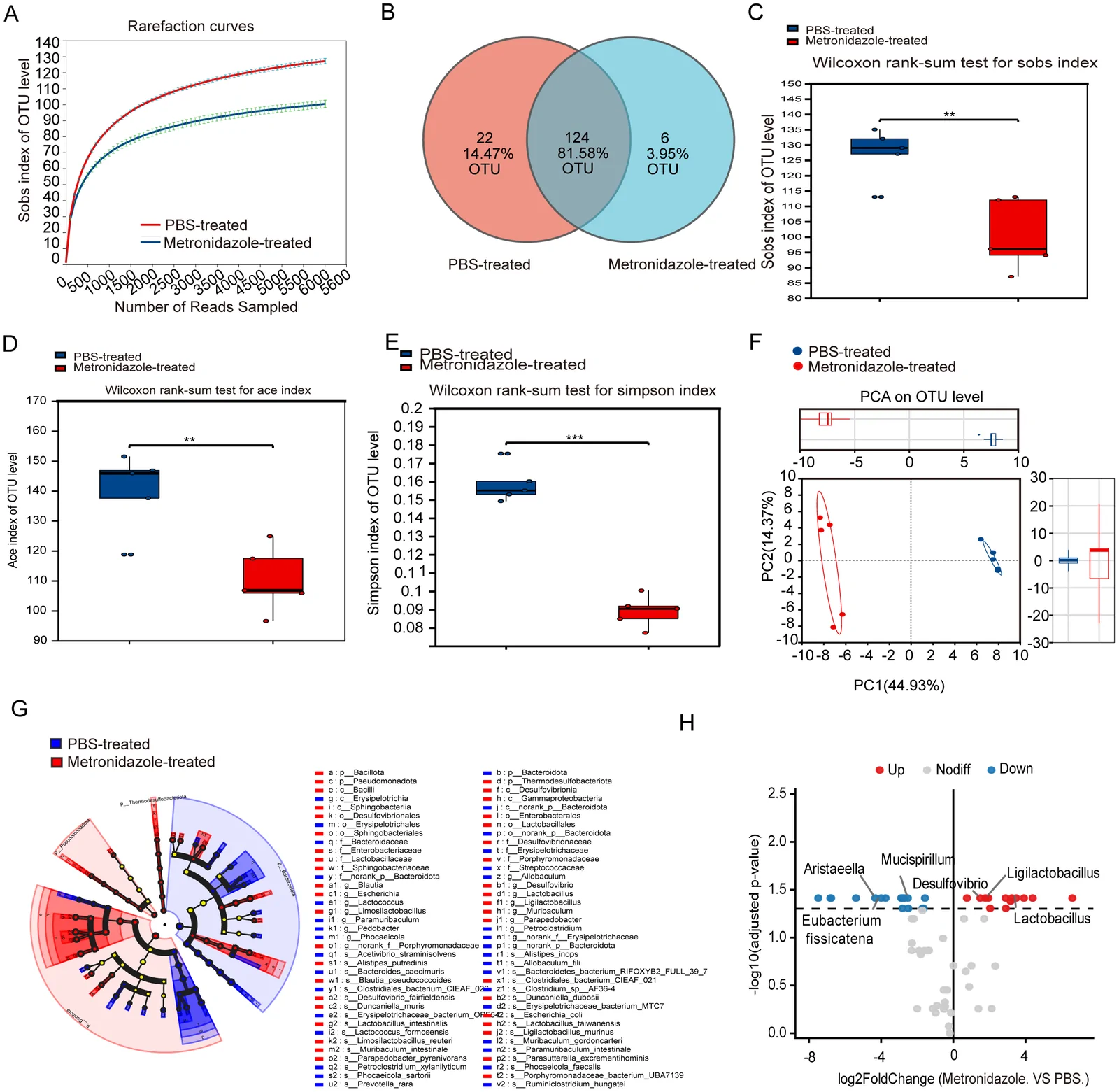
Metronidazole-mediated remodeling of gut microbial diversity and community structure *in vivo*. **(A)** Rarefaction curve based on observed OTUs indicate sufficient sequencing depth and coverage in both metronidazole-treated and PBS-treated groups. **(B)** The Venn plots reveal the number of shared and unique OTUs between metronidazole-treated and PBS-treated mice. **(C–E)** Alpha diversity, including Sobs index, ACE index and Simpson, reveals significant differences in microbial diversity and richness between metronidazole-treated and PBS-treated. **(F)** Principal component analysis (PCA) based on OTU abundance profiles showing distinct clustering of gut microbial communities between the metronidazole-treated or PBS-treated groups. **(G)** LEfSe analysis identifies differentially enriched bacterial taxa between metronidazole-treated and PBS-treated group. **(H)** Genus-level volcano plot of gut bacterial taxa altered by metronidazole treatment. Red and blue dots indicate taxa enriched in metronidazole-treated and PBS-treated mice, respectively.

To determine whether metronidazole pretreatment altered the gut microbial community, fecal samples were collected on day 3 after transplantation and subjected to 16S rRNA gene sequencing. Rarefaction curves approached saturation in both groups, indicating adequate sequencing depth and sufficient coverage of microbial diversity (**Figure 2A**). A total of 152 OTUs were identified across all samples of which 124 OTUs were shared between the metronidazole-treated and PBS-treated groups. In contrast, 22 and 6 OTUs were uniquely detected in the PBS-treated and metronidazole-treated groups, respectively (**Figure 2B**). These findings indicate that the two groups contained distinct group-specific OTUs, with fewer unique OTUs detected in the metronidazole-treated group. We next assessed within-sample diversity. Richness-related indices, including Sobs (P = 0.003) and Ace (P = 0.004), were significantly lower in the metronidazole-treated group than in the PBS-treated group (**Figures 2C,D**), whereas the Chao index did not differ significantly between groups (P = 0.06, **Supplementary Figure 2A**). Moreover, the Simpson index was significantly increased following metronidazole treatment (*P* = 2.631e-6), whereas no significant differences were observed for the Shannon (*P* = 0.12) or Coverage indices (*P* = 0.18) (**Figure 2E**; **Supplementary Figures 2B,C**). Collectively, these findings indicate that metronidazole treatment was associated with altered community richness and dominance structure, whereas the overall diversity pattern showed only partial changes. Principal component analysis (PCA) on OTU-based community profiles demonstrated a distinct clustering of metronidazole-treated and PBS-treated groups, with PC1 and PC2 accounting for 44.93% and 7.99% of the total variance, respectively (**Figure 2F**). Beta-diversity was further assessed using Bray–Curtis-based PCoA and PERMANOVA. Consistent with the PCA results, PCoA showed group-level separation, and PERMANOVA confirmed a significant difference in microbial community structure between the two groups (**Supplementary Figure 3**; **Supplementary Table 5**, F = 56.999, R² = 0.877, P = 0.005). These findings indicate that metronidazole pretreatment substantially reshaped the gut microbial community. LEfSe analysis further identified distinct microbial signatures differentiating the metronidazole-treated and PBS-treated groups. Specifically, *Pseudomonadota*- and *Thermodesulfobacteriota*-associated lineages were preferentially enriched in metronidazole-treated mice, whereas *Bacteroidota*-associated taxa were predominantly enriched in PBS-treated controls (**Figure 2G**). Consistently, genus-level volcano analysis highlighted taxa that differed between the two groups after metronidazole treatment. Among these, *Lactobacillus*, *Ligilactobacillus* and *Desulfovibrio* showed increased relative abundance in metronidazole-treated mice, whereas *Aristaeella*, *Mucispirillum* and *Eubacterium fissicatena* were reduced after metronidazole treatment (**Figure 2H**; **Supplementary Table 6**). Collectively, these exploratory findings indicate that metronidazole pretreatment was associated with marked changes in gut microbial composition in recipient mice.

### 3.3 Mendelian randomization analysis of gut microbial taxa associated with allograft rejection

**Figure 3:**
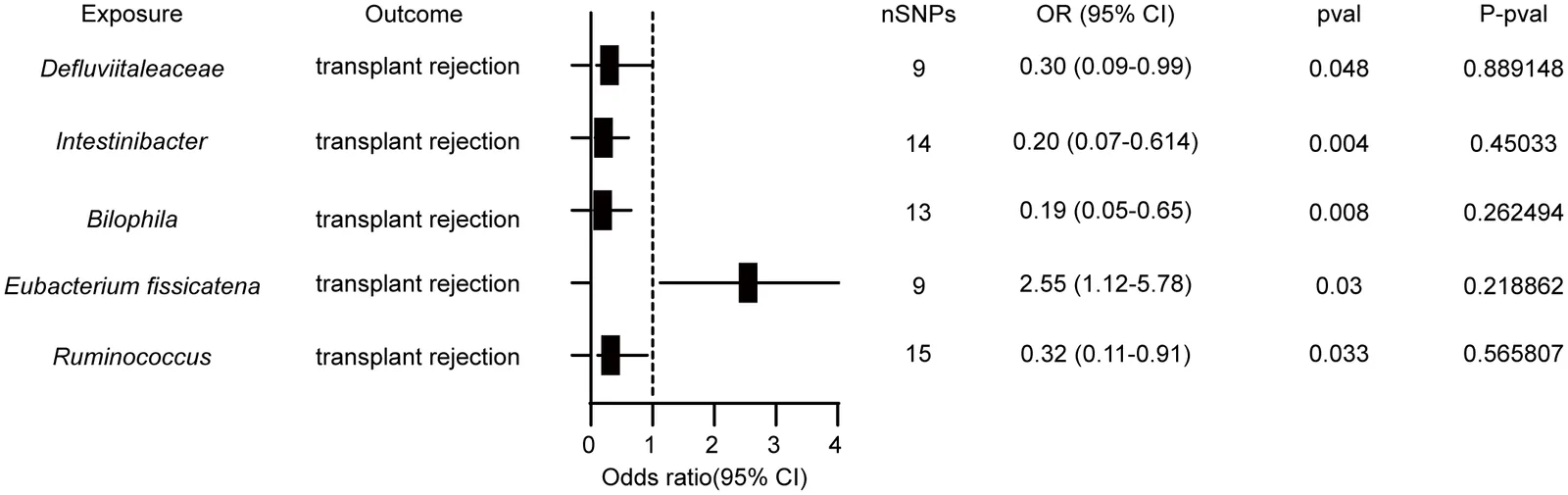
Results in the forward MR. The forest plot shows MR estimates for the associations between genetically predicted microbial abundance and the composite outcome of transplant failure or rejection. Data are expressed as an odds ratio (OR) with corresponding 95% confidence interval (CI). P-pval, horizontal pleiotropy p value.

Separately from the murine 16S rRNA sequencing analysis, we conducted an exploratory two-sample MR analysis using human GWAS data from MiBioGen and FinnGen. This analysis aimed to evaluate whether genetically predicted variation in human gut microbial taxa was associated with transplant rejection risk. MR analyses were conducted across multiple taxonomic levels, including order, family, and genus. Associations between the relative abundance of gut microbial taxa and transplant rejection were evaluated using four complementary MR methods, including IVW, MR-Egger, weighted median and weighted mode approaches. Several microbial taxa showed associations with transplant rejection risk across the MR analyses. Specifically, the family *Defluviitaleaceae*, and genera *Intestinibacter*, *Bilophila* and *Ruminococcus* were consistently associated with a reduced risk of transplant rejection, whereas the genus *Eubacterium fissicatena* was associated with an increased risk of transplant rejection (**Supplementary Table 7**; **Figure 3**). Using IVW as the primary estimator, genetically predicted higher abundances of *Intestinibacter* (OR = 0.2, 95% CI: 0.07–0.61; *P* = 0.004), *Bilophila* (OR = 0.19, 95% CI: 0.05–0.65; *P* = 0.008), and *Ruminococcus* (OR = 0.32, 95% CI: 0.11–0.91; *P* = 0.033) were associated with a reduced risk of transplant rejection, and *Defluviitaleaceae* showed a similar direction of effect (OR = 0.30, 95% CI: 0.09–0.99; *P* = 0.048). As shown in **Supplementary Figures 4A–E**, genetically predicted higher abundance of *Eubacterium fissicatena* was associated with an increased risk of transplant rejection (OR = 2.55, 95% CI: 1.12–5.78; *P* = 0.03). Scatterplots depicting the associations between individual SNP instruments and transplant rejection for microbial taxa with significant MR effects (P < 0.05) are shown in **Supplementary Figures 5A–E**.

To evaluate the reliability and stability of MR results and to assess potential biases influenced by individual instrumental variables, we performed a series of sensitivity analyses, including heterogeneity testing, horizontal pleiotropy assessment, and leave-one-out analyses. No evidence of heterogeneity was observed for *Defluviitaleaceae (P* = 0.908*)*, *Intestinibacter (P* = 0.927*)*, *Bilophila (P* = 0.533*)*, or *Eubacterium fissicatena (P* = 0.847*)* based on Cochran’s Q statistic. A moderate degree of heterogeneity was observed only for *Ruminococcus* based on the random-effects model (*P* = 0.047; I² = 47%) (**Supplementary Table 8**). Nevertheless, MR-Egger intercept analyses showed no evidence of horizontal pleiotropy across all examined taxa, suggesting that the observed associations, including the estimate for *Ruminococcus*, were unlikely to be driven by directional pleiotropic effects. For microbial taxa exhibiting significant IVW associations (*P* < 0.05), leave-one-out analyses were conducted to assess whether individual SNP instruments disproportionately influenced the MR estimates. Sequential exclusion of individual SNP instruments did not materially alter the magnitude or direction of the MR estimates, indicating that the observed associations were not driven by any single variant. Sensitivity analyses showed no evidence of substantial heterogeneity or directional pleiotropy. The detailed results of the sensitivity analyses, heterogeneity tests, and pleiotropy evaluations are provided in **Supplementary Tables 9**-**11**. Collectively, the MR analysis identified several microbial taxa showing nominal associations with allograft rejection, which were considered exploratory candidates for further evaluation.

### 3.4 Biological annotation highlights glycosylation-related hub genes linking gut microbial taxa to transplant rejection

We next conducted an exploratory comparison between the murine 16S rRNA sequencing dataset and the human MR results. Among the taxa prioritized by the human MR analysis, *Eubacterium fissicatena* and *Ruminococcus* were also detected in the murine dataset (**Supplementary Table 6**). *Eubacterium fissicatena* showed a significant reduction (adjusted *P* = 0.03869) after metronidazole treatment, whereas *Ruminococcus* was detectable in both groups but was not significantly altered by metronidazole intervention (adjusted *P* = 0.6139). These MR-prioritized taxa, which were also detectable in the murine dataset, were selected for downstream SNP-based biological annotation. Corresponding variants from the human microbiome GWAS were mapped to nearby genes and further evaluated using protein–protein interaction (PPI) and functional enrichment analyses.

**Figure 4:**
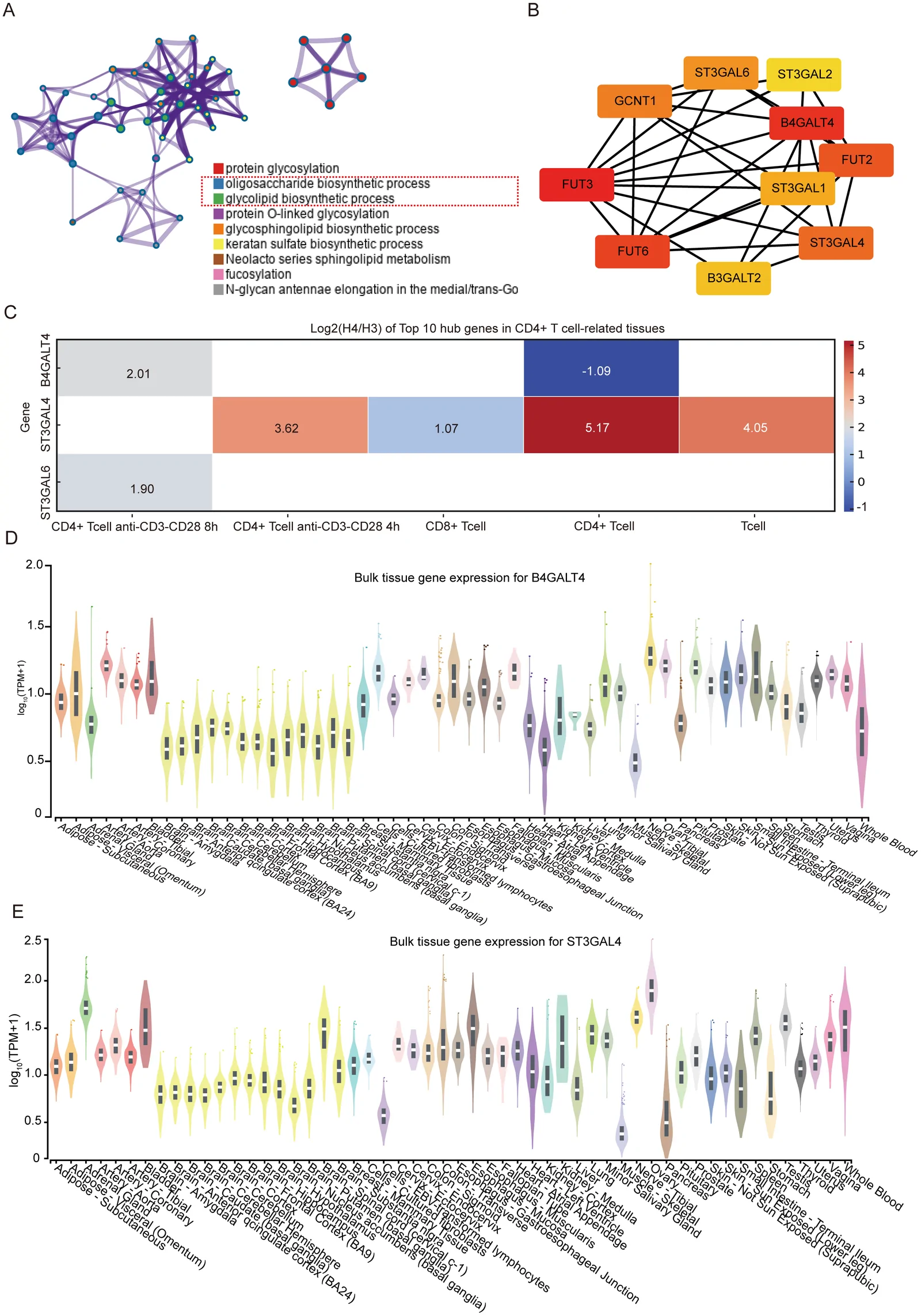
Exploratory biological annotation identifies glycosylation-related host candidate genes. **(A)** Protein-protein interaction (PPI) network of top 12 glycosylation-related hub genes identified using STRING and visualized with Cytoscape. Node color indicates degree centrality, with red representing higher connectivity within the network. **(B)** Functional enrichment analysis of the PPI network using Gene Ontology (GO) biological process. Each node represents a GO term, and edge thickness corresponds to the degree of gene overlap. Enriched processes are predominantly related to protein glycosylation, oligosaccharide biosynthetic processes, and protein O-linked glycosylation. **(C)** Relative expression pattern of B4GALT4 and ST3GAL4 across CD4^+^ T cell-related datasets from GEO. The heatmap shows Log_2_ (H4/H3) expression ratios across different T-cell subset and activation conditions. **(D, E)** Tissue-wide expression profiles of B4GALT4 **(D)** and ST3GAL4 **(E)** based on data from the Genotype-Tissue Expression project (GTEx). Violin plots show normalized gene expression in immune-related tissues.

Using STRING (v11.5) and Cytoscape, we identified several putative hub genes, including FUT3, B4GALT4, FUT6, and ST3GAL1 (**Figure 4A**). We next examined the functional organization of these candidate genes using GO-based analysis, which showed that several enriched biological process clusters were related to protein glycosylation, glycosphingolipid biosynthesis, and fucosylation (**Figure 4B**). These findings suggest that host genetic loci linked to the prioritized microbial taxa converge on glycosylation-related pathways. Glycosylation is a key post-translational modification involved in CD4^+^ T-cell function and immune ligand–receptor interactions. We therefore examined the expression of glycosylation-related candidate genes across CD4^+^ T-cell subsets and activation states (Rudd et al., 2001). Among the glycosylation-related candidates, B4GALT4, ST3GAL6, and ST3GAL4 were selected for immune-context expression analysis based on their annotation profiles and potential relevance to T-cell activation. The log_2_-normalized expression levels of these genes were then assessed across different CD4^+^ T-cell activation states. Notably, ST3GAL4 and B4GALT4 showed higher expression in activated CD4^+^ T cells compared with resting conditions, indicating an activation-dependent transcriptional upregulation (**Figure 4C**). Additionally, GTEx data showed detectable expression of ST3GAL4 and B4GALT4 in immune-relevant tissues, including the spleen and colon (**Figures 4D,E**). Taken together, these integrative annotation analyses highlighted glycosylation-related host pathways and prioritized ST3GAL4 and B4GALT4 as candidate genes for subsequent expression assessment.

### 3.5 Metronidazole reshapes the post-transplant immune microenvironment

**Figure 5:**
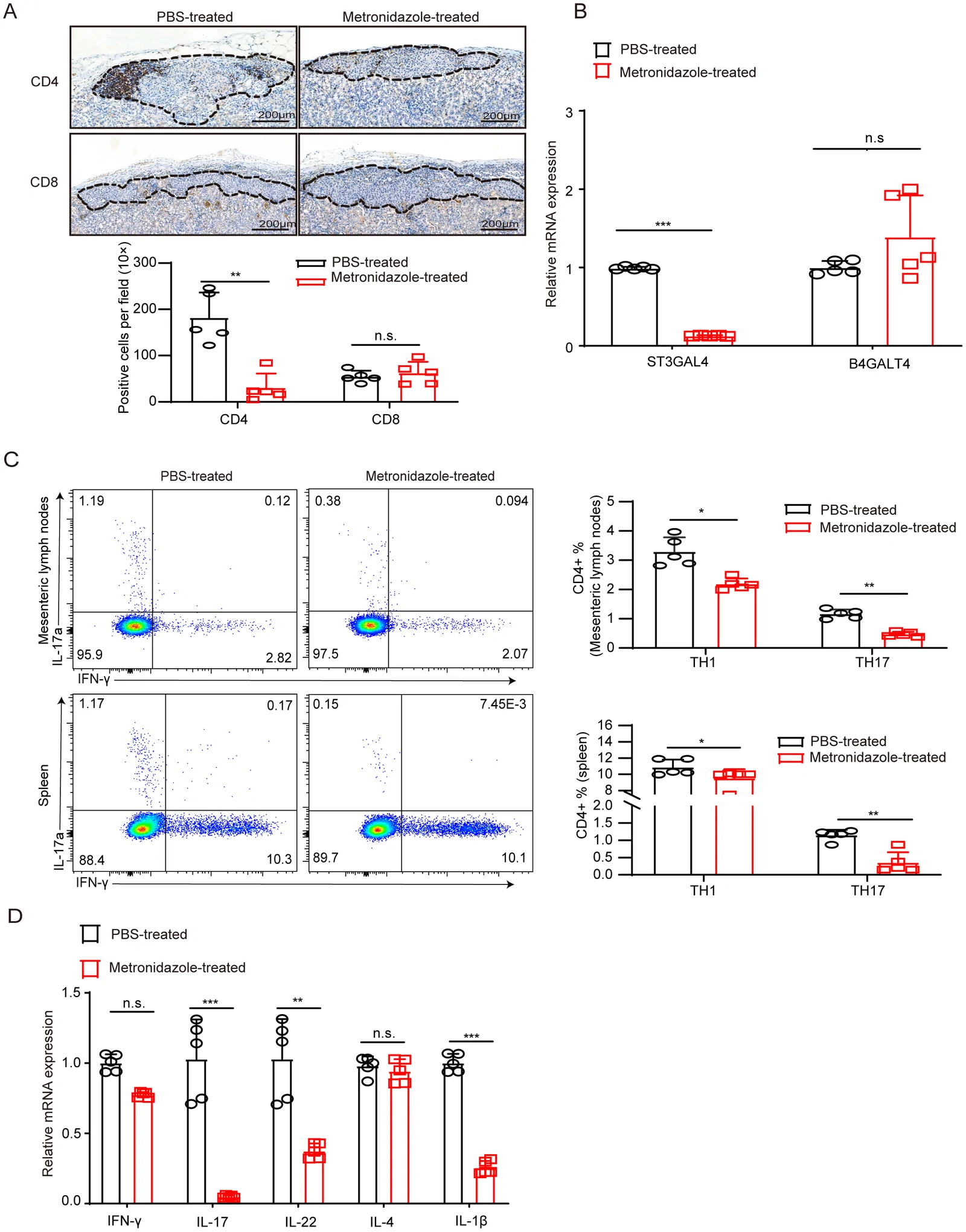
Metronidazole pretreatment is associated with altered graft and systemic immune responses after transplantation. **(A)** Representative immunohistochemical images and quantification of CD4^+^ and CD8^+^ T-cell infiltration in islet grafts. Data are expressed as the mean number of positive cells per field, with each point representing one recipient mouse (n = 5 per group). Scale bars, 200 μm. **(B)** Relative expression levels of glycosylation-related genes ST3GAL4 and B4GALT4 in islet graft tissues from metronidazole-treated and PBS-treated recipient mice. **(C)** Representative flow-cytometry plots and corresponding quantification of Th1 (CD4^+^IFN-γ^+^) and Th17 (CD4^+^IL-17A^+^) cells in the mesenteric lymph nodes and spleen on day 7 after transplantation. A total of 1 × 10^6^ cells per sample were analyzed. Frequencies are expressed as percentages of CD4^+^ T cells, and each point represents one recipient mouse (n = 5 per group). **(D)** Relative mRNA expression levels of selected genes in recipient mice from metronidazole-treated and PBS-treated groups after islet transplantation, as determined by qRT–PCR. Data information: In Panel **(A)**, CD4^+^ and CD8^+^ positive cells were counted in randomly selected fields and averaged for each graft. Panel **(B)** shows the relative mRNA expression levels normalized to that of the housekeeping gene GAPDH; the results are presented as the mean ± SD, and each point in Panels **(A–D)** represents one recipient mouse (n = 5 per group). Statistical comparisons were performed using an unpaired two-tailed Student’s t-test. *p < 0.05, **p < 0.01, ***p < 0.001, p values >0.05 were considered nonsignificant (n.s.).

Additionally, functional prediction analysis based on 16S rRNA sequencing data integrated with KEGG annotations revealed significant enrichment of pathways related to inflammatory and immune processes (**Supplementary Figure 6**), supporting a potential link between metronidazole-induced microbiota remodeling and immune regulation. To further evaluate the local immune microenvironment after transplantation, immunohistochemical analysis was performed around the islet grafts. Results demonstrated a reduction in CD4^+^ T-cell infiltration surrounding the islet allografts in metronidazole-treated mice, whereas CD8^+^ T-cell infiltration remained largely unchanged (**Figure 5A**). Consistent with the biological annotation analyses, graft ST3GAL4 expression was significantly lower in metronidazole-treated mice than in PBS-treated mice (**Figure 5B**). Previous studies have reported that *St3gal4* deficiency markedly reduces the expression of IFN-γ and IL-17A, which drive Th1 and Th17 cell expansion and promote the development of colitis (Kurakevich et al., 2013). We next assessed whether these changes were accompanied by alterations in Th17-related immune responses. Flow cytometric analysis showed that the frequencies of Th17 cells in both mesenteric lymph nodes and spleen were reduced in metronidazole-treated mice compared with PBS-treated controls (**Figure 5C**). Consistently, cytokine transcript analysis of colon tissues was performed and results demonstrated that metronidazole treatment significantly reduced the expression of IL-17A, IL-1β, and IL-22, while IFN-γ and IL-4 transcript levels remained unchanged (**Figure 5D**). Taken together, these findings indicate that metronidazole treatment is associated with reduced graft ST3GAL4 expression, decreased CD4^+^ T-cell infiltration, and attenuation of Th17-related inflammatory responses.

## 4 Discussion

Transplant rejection remains mechanistically incompletely understood, and the contribution of the microbiota–immune axis to alloimmune injury is only beginning to be defined. In this study, metronidazole pretreatment prolonged islet allograft survival time and substantially reshaped gut microbial composition. MR analysis provided independent human genetic evidence for the association between several anaerobe-associated gut microbial taxa and transplant rejection risk. Meanwhile, integrative annotation prioritized glycosylation-related host pathways, particularly ST3GAL4. In parallel, metronidazole-mediated microbiota remodeling was accompanied by reduced ST3GAL4 expression in the graft. Metronidazole pretreatment also decreased CD4^+^ T-cell infiltration and attenuated Th17-related inflammatory responses after transplantation. Together, these findings suggest that anaerobe-associated gut microbial changes were accompanied by altered alloimmune responses after transplantation.

Previous clinical investigations have reported markedly reduced gut microbial diversity in lung transplant recipients with allograft rejection, accompanied by a substantial decrease in the number of detected species. By contrast, tolerant lung transplant recipients retain *Firmicutes* and *Bacteroidetes* as the dominant phyla, resembling the microbial configuration observed in healthy individuals (Wu et al., 2023). Because many *Firmicutes and Bacteroidetes* are obligate anaerobes, their depletion during rejection suggests that anaerobic gut communities may be affected in transplant-associated dysbiosis. In the present study, metronidazole pretreatment broadly remodeled the gut microbial community and was accompanied by prolonged graft survival (**Figures 1**, **2**). 16S rRNA sequencing revealed structural alterations involving *Lactobacillus*, *Ligilactobacillus*, *Desulfovibrio, Aristaeella, Mucispirillum and Eubacteriales*-associated taxa, further highlighting changes in anaerobe-associated microbial composition following metronidazole treatment (**Figure 2**). The exploratory human MR analysis independently prioritized taxa showing nominal associations with the composite transplant outcome, including *Intestinibacter*, *Bilophila*, *Ruminococcus*, *Defluviitaleaceae and Eubacterium fissicatena*, many of which shared ecological features related to anaerobic metabolism (**Figure 3**). These observations support further investigation of anaerobe-associated microbial changes in transplantation. Although metronidazole broadly remodels the gut microbial community, its effect on prolonged graft survival cannot be attributed to changes in any single bacterial taxon based on the current data. *Eubacterium fissicatena* therefore warrants further investigation among the MR-prioritized taxa. A higher genetically predicted abundance of *Eubacterium fissicatena* was associated with an increased risk of transplant rejection, indicating that microbial features related to this taxon serve as potential biomarkers of transplant rejection. However, the biological characteristics and immunological relevance of *Eubacterium fissicatena* in transplant settings remain poorly defined and the current data do not establish a direct functional role for this bacterium in graft injury. Given that metronidazole broadly remodels the gut microbial community, future studies using *E. fissicatena* reconstitution in metronidazole-treated or antibiotic-depleted recipients will be needed to determine whether this bacterium directly accelerates graft rejection.

Sialyltransferases (STs) catalyze terminal sialylation of membrane-bound and secreted proteins, a dynamic glycosylation process involved in the regulation of protein function and cellular signaling (Pinho and Reis, 2015; Hodgson et al., 2024). Among these enzymes, ST3GAL4 has been implicated in cancer biology, including tumor proliferation, metastatic dissemination and poor clinical outcomes across multiple cancer types, highlighting its relevance in glycosylation-dependent pathophysiological processes (Wu et al., 2024; Yang et al., 2025). In the present study, integrative biological annotation highlighted glycosylation-related pathways among the host genes mapped from microbiota-associated genetic signals (**Figures 4**, **5**). Notably, ST3GAL4 expression was lower in grafts from metronidazole-treated recipients, alongside the prolonged allograft survival observed in this group (**Figure 5B**). These findings raise the possibility that microbiota-associated signals may intersect with glycosylation programs upstream of alloimmune injury, although mechanistic studies are needed to determine whether specific microbial cues influence ST3GAL4-dependent glycosylation pathways in transplant immunity.

Microbiota-derived signals are important regulators of host immune homeostasis (Ohtani, 2015; Qin et al., 2021). For example, germ-free animals exhibit markedly reduced immune responses compared with conventionally raised counterparts when exposed to the same viral infection, underscoring the necessity of commensal microbes in immune system development (Ganal et al., 2012). Similarly, *Fusobacterium nucleatum* suppresses anti-tumor T cell responses, thereby promoting the progression of colorectal cancer (Zhou et al., 2021). In transplant medicine, disruption of microbial community structure has been associated with persistent immunological alterations and increased rejection risk (Wu et al., 2023). How anaerobe-associated gut microbial changes shape transplant immunity remains unclear. In our study, annotation analyses showed that microbiota-associated signals converged on glycosylation-related pathways. Given the role of glycosylation in T-cell activation and cytokine signaling, we speculate that glycosylation-related host programs may warrant investigation as a candidate pathway between anaerobe-associated gut microbial changes and transplant immunity (Pan et al., 2025). Consistent with this view, several glycosylation-related hub genes were enriched in immune-relevant tissues, and public immune-cell transcriptomic datasets further showed dynamic expression across different CD4^+^ T-cell activation states (**Figures 4C–E**). These findings provided a rationale for further examining glycosylation-related immune changes in the transplantation. To further characterize immune changes associated with microbiota modulation *in vivo*, immunohistochemical analysis demonstrated reduced CD4^+^ T-cell infiltration within the islet allograft area in metronidazole-treated mice, whereas CD8^+^ T-cell infiltration remained largely unchanged (**Figure 5A**). Given the established role of CD4^+^ T-cell subset imbalance, particularly a Th17-skewed Th17/Treg axis, in systemic inflammation and allograft dysfunction (Huang et al., 2023), we next assessed Th17-cell responses. Flow-cytometric analysis demonstrated that metronidazole-mediated gut microbiota remodeling was associated with diminished Th17-cell frequencies in the mesenteric lymph nodes and spleen after transplantation (**Figure 5C**). Together, these findings indicate that targeted modulation of gut microbiota composition may shape the immune microenvironment after transplantation, particularly through reduced CD4^+^ T-cell infiltration and attenuated Th17-cell responses.

In conclusion, this study provides exploratory cross-dataset evidence supporting the potential relevance of anaerobe-associated gut microbiota to graft dysfunction following transplantation. Furthermore, These findings support further investigation of gut microbiota modulation in transplantation.

## 5 Limitations

Several limitations should be acknowledged. For the MR analysis, we used a suggestive SNP threshold of *P < *1 × 10^-5^ to retain sufficient instruments for gut microbial taxa; however, this threshold is less stringent than genome-wide significance and may introduce uncertainty related to instrument strength (Kurilshikov et al., 2021; Liu et al., 2022).

The murine 16S and human MR analyses were performed independently. Although taxonomic overlap provided a basis for candidate prioritization, shared taxa do not necessarily represent species-, strain-, or functionally conserved microbial features across hosts. Therefore, the integrated analysis should be interpreted as hypothesis-generating rather than as evidence that specific taxa directly mediate metronidazole-associated graft protection. Interpretation of the MR findings is further limited by the heterogeneous FinnGen transplant endpoint, the lack of organ- and rejection-specific information, and the relatively small number of cases (124 cases and 199,271 controls). Together with the use of a suggestive instrument-selection threshold and the lack of definitive multiple-testing support, these factors require cautious interpretation of the identified taxa as exploratory candidates. Future studies using organ-specific cohorts and functional microbial validation will be needed to determine their relevance to islet allograft rejection. Daily water consumption was not measured, and the relatively small cohort (n = 5 individually housed mice per group) limited the precision and power of the analyses. Although individual housing minimized microbial exchange and within-cage clustering, the microbiome findings require validation in larger independent cohorts with quantitative monitoring of water intake.

Finally, positional mapping, PPI network analysis, and graft ST3GAL4 expression changes nominated glycosylation-related host pathways, but they do not establish functional causality. Future studies involving taxon-specific reconstitution, ST3GAL4 functional manipulation, and glycosylation profiling will be required to clarify the underlying mechanisms.

## Data Availability

The raw 16S rRNA sequencing data generated in this study have been deposited in the NCBI Sequence Read Archive (SRA) under BioProject accession number PRJNA1509593. The corresponding SRA accession numbers are SRR40073369–SRR40073378. The data are available at https://www.ncbi.nlm.nih.gov/bioproject/PRJNA1509593.
