# Supplementary material for: Metronidazole-mediated gut anaerobe remodeling is associated with transplant rejection
Source: Front Cell Infect Microbiol. 2026 Aug 18;16:1874387. doi: 10.3389/fcimb.2026.1874387 (PMC13529449; doi:10.3389/fcimb.2026.1874387)
Supplement: Supplementary file 1 [file DataSheet1.docx]

**Supplementary Figure1**

**Schematic representation of a two-sample randomization analysis.**

MR was used to evaluate the causal links between 198 microbial taxa and transplant rejection. Four key assumptions of MR: (1) The genetic variant is strongly related to the exposure (Step 1); (2) It is independent of any confounders affecting the exposure-outcome relationship (Step 1); (3) Its influence on the outcome is exclusively attributed to the exposure, barring potential horizontal pleiotropy.

**Supplementary Figure 2**

**Metronidazole treatment alters gut microbiota α-diversity at the OTU level.**
(A) Coverage index at the OTU level in metronidazole-treated and PBS-treated groups. (B) Chao richness index at the OTU level in metronidazole-treated and PBS-treated groups. (C) Shannon diversity index at the OTU level in metronidazole-treated and PBS-treated groups.

**Supplementary Figure 3**

**Bray–Curtis-based PCoA analysis of gut microbial community structure.**

Principal coordinate analysis (PCoA) was performed using the Bray–Curtis distance matrix calculated from OTU-level abundance profiles. Each point represents an individual mouse sample. PBS-treated mice are shown in blue, and metronidazole-treated mice are shown in red. The first two principal coordinates explained 87.29% and 5.4% of the total variation, respectively. PERMANOVA confirmed a significant difference in microbial community structure between the two groups (F = 56.999, R² = 0.877, P = 0.005).

**Supplementary Figure 4**

**Leave-one-out plots for MR results.**

Forest plot of causal estimates omitting each variant in turn.

**Supplementary Figure 5**

**MR scatter plots.**

Scatterplot of SNP potential effects on microbial taxa. (A)-(D) represent genus-level, including *Bilophila* (A), Intestinibacter (B), *Ruminococcus* (C) *and Eubacterium fissicatena* (D); (E) represent the *family-level Defluviitaleaceae.* The slope of each line corresponding to the causal effect estimates derived from different Mendelian randomization methods, including inverse-variance weighted, MR-Egger, weighted median, and weighted mode.

**Supplementary Figure 6**

**Predicted functional profiles of gut microbiota after metronidazole treatment**

(A) Heatmap of predicted KEGG functional pathways based on 16S rRNA sequencing data. Color intensity represents the relative abundance of predicted gene counts across major functional categories.
