# Supplementary material for: Metronidazole-mediated gut anaerobe remodeling is associated with transplant rejection
Source: Front Cell Infect Microbiol. 2026 Aug 18;16:1874387. doi: 10.3389/fcimb.2026.1874387 (PMC13529449; doi:10.3389/fcimb.2026.1874387)
Supplement: Supplementary file 2 [file Table2.docx]

**STROBE-MR checklist of recommended items to address in reports of Mendelian randomization studies**^1^ ^2^

| **Item No.** | **Section** | **Checklist item** | **Page No.** | **Relevant text from manuscript** |
| --- | --- | --- | --- | --- |
| 1 | **TITLE and ABSTRACT** | The study title and/or abstract should explicitly indicate the use of Mendelian randomization (MR) when it represents a primary study design | Article | Systematic Genome-Wide Mendelian Randomization Identifies Causal Gut Microbiota and Therapeutic Targets for Transplant Rejection |
|  | **INTRODUCTION** |  |  |  |
| 2 | **Background** | Provide a clear scientific background and rationale for the study, explicitly defining the exposure of interest and assessing the biological plausibility of a causal relationship between the exposure and the outcome. Furthermore, explain why Mendelian randomization (MR) is particularly well suited to address this causal research question | Article | Mendelian randomization (MR) is an epidemiological approach that leverages genetic variants as instrumental variables (IVs) to infer causal relationships between risk factors and outcomes. Because genetic variants are randomly allocated at conception and are largely independent of environmental and behavioural confounders, MR can reduce bias from confounding and reverse causation, thereby providing more robust evidence for causal inference. |
| 3 | **Objectives** | Clearly state the specific objectives of the study, including any pre-specified causal hypotheses. Indicate that Mendelian randomization (MR) is a methodological approach that, under specific assumptions, aims to estimate causal effects between exposures and outcomes. | Article | The objective of this study was to investigate the influence of the gut microbiota on outcomes following organ and tissue transplantation. Using a two-sample Mendelian randomization (MR) framework, we evaluated the causal effects of 211 gut microbiota–related factors on transplantation outcomes. The findings of this study may provide novel insights into modifiable microbial determinants in transplant recipients and contribute to the development of evidence-based strategies to improve post-transplant outcomes. |
|  | **METHODS** |  |  |  |
| 4 | **Study design and data sources** | Present the key elements of the study design early in the manuscript. Consider including a table summarizing the data sources used across all phases of the study. For each data source contributing to the analysis, provide a detailed description of the following elements: |  |  |
|  | a) | Setting: Describe the study design and the underlying study population, where applicable. Specify the study setting, geographic locations, and relevant time periods, including recruitment, exposure assessment, follow-up, and data collection, when available. | Article | 2.1-2.2 Study design and data sources |
|  | b) | Participants: Define the eligibility criteria and describe the sources and methods used to select participants. Report the sample size and indicate whether any power or sample size calculations were conducted prior to the primary analysis. | Article | Table 1 |
|  | c) | Describe the measurement, quality control procedures, and selection criteria applied to the genetic variants used in the analysis. | Article | 2.3 Instrumental variable selection |
|  | d) | For each exposure, outcome, and other relevant variables, describe the methods of assessment and, where applicable, the diagnostic criteria used to define diseases or clinical endpoints. | Article | 2.3 Instrumental variable selection |
|  | e) | Provide details of ethics committee approval and procedures for obtaining informed consent from participants, where relevant. | / | / |
| 5 | **Assumptions** | Explicitly state the three-core instrumental variable (IV) assumptions underlying the primary analysis—relevance, independence, and exclusion restriction—as well as any additional assumptions relevant to sensitivity or supplementary analyses. | Article | 2.10 Statistical analyses |
| 6 | **Statistical methods: main analysis** | Describe the statistical methods and summary statistics used in the analyses. |  |  |
|  | a) | Quantitative variables: specify how quantitative variables were handled, including their scale, units, transformations (if any), and the statistical models applied. | Article | 2.3 Instrumental variable selection |
|  | b) | Genetic variants: describe how genetic variants were incorporated into the analyses, including quality control procedures and, where applicable, how variant weights were selected. | Article | 2.3 Instrumental variable selection |
|  | c) | Mendelian randomization methods: specify the Mendelian randomization (MR) estimator(s) used (e.g., Wald ratio, inverse-variance weighted method, two-stage least squares) and the corresponding statistical measures. Detail the covariates included in the models and, in the case of two-sample MR, indicate whether consistent covariate adjustment was applied across both samples. | Article | 2.10 Statistical analyses |
|  | d) | Missing data: explain how missing data were handled in the analyses. | / | / |
|  | e) | Multiple testing: if applicable, describe the approach used to address multiple testing. | / | / |
| 7 | **Assessment of assumptions** | Describe any methods, prior evidence, or domain knowledge used to assess the validity of the instrumental variable assumptions and to justify their plausibility | Article | 2.10 Statistical analyses |
| 8 | **Sensitivity analyses and additional analyses** | Describe any sensitivity analyses or additional analyses performed, such as comparisons of effect estimates across different MR approaches, independent replication, bias-analytic techniques, validation of genetic instruments, or simulation analyses, where applicable | Article | 2.10 Statistical analyses |
| 9 | **Software and pre-registration** |  |  |  |
|  | a) | Specify the statistical software and packages used for all analyses, including version numbers and relevant settings | Article | 2.10 Statistical analyses |
|  | b) | State whether the study protocol and analytical plan were pre-registered, and if so, provide details on the registration platform, timing, and registration identifier | Article | 2.10 Statistical analyses |
|  | **RESULTS** |  |  |  |
| 10 | **Descriptive data** |  |  |  |
|  | a) | Report the number of individuals included at each stage of the study and provide reasons for exclusion. The use of a flow diagram is recommended to enhance clarity and transparency. | Article | Figure 1 |
|  | b) | Present summary statistics for phenotypic exposures, outcomes, and other relevant variables, such as means, standard deviations, and proportions, as appropriate. | Article | Table 1 |
|  | c) | If the data sources include meta-analyses of multiple studies, report assessments of heterogeneity across contributing studies. | / | / |
|  | d) | For two-sample MR analyses:  i. Provide justification for the comparability of genetic variant–exposure associations between the exposure and outcome datasets.  ii. Report the extent of sample overlap between the exposure and outcome studies, including the number or proportion of overlapping individuals, if applicable. | Article | 2.3 Instrumental variable selection |
| 11 | **Main results** |  |  |  |
|  | a) | Report the associations between each genetic variant and the exposure, as well as between each genetic variant and the outcome, preferably expressed on an interpretable scale | Supplementary | Supplementary Table S5 |
|  | b) | Present the Mendelian randomization (MR) estimates for the relationship between the exposure and the outcome, together with corresponding measures of uncertainty, such as confidence intervals, on an interpretable scale (e.g., odds ratios or relative risks per standard deviation increase in the exposure) | Supplementary | Supplementary Table S5 |
|  | c) | Where relevant, consider translating relative risk estimates into absolute risk differences over a clinically meaningful time period | / | / |
|  | d) | Use appropriate graphical displays to visualize the results, such as forest plots or scatter plots illustrating the associations between genetic variants and the outcome versus the exposure | Article | Figure 2 & Figure 4 |
| 12 | **Assessment of assumptions** |  |  |  |
|  | a) | Report the results of assessments evaluating the validity of the instrumental variable assumptions underlying the Mendelian randomization analysis | / | / |
|  | b) | Report any additional relevant statistical measures, such as assessments of heterogeneity across genetic variants (e.g. Cochran’s *Q* statistic or *E*-values), where applicable | Supplementary | Supplementary Table S5 |
| 13 | **Sensitivity analyses and additional analyses** |  |  |  |
|  | a) | Report any sensitivity analyses performed to evaluate the robustness of the primary results to potential violations of the Mendelian randomization assumptions | / | Figure 2 |
|  | b) | Report the results of any additional sensitivity or supplementary analyses conducted to further assess the stability of the findings | / | Figure 2 |
|  | c) | Report any analyses undertaken to assess the direction of the causal relationship, such as bidirectional Mendelian randomization | / | Figure 2 |
|  | d) | When relevant, report and compare the MR-derived estimates with results obtained from non-MR analytical approaches | / | / |
|  | e) | Consider presenting additional plots to visualize robustness and influence diagnostics, such as leave-one-out analyses or other sensitivity plots | Supplementary | Supplementary Table 4-7 |
|  | **DISCUSSION** |  |  |  |
| 14 | **Key results** | Summarize key results with reference to study objectives | Article | In summary, our two-sample Mendelian randomization study identified significant associations between genetically predicted gut microbial and organ and tissue transplantation. Specifically, several microbial taxa were causally associated with a reduced risk of transplant rejection, whereas gut microbial exhibited risk-enhancing effects. These findings provide evidence that alterations in the gut microbiota may play a causal role in shaping transplant outcomes. However, no causal associations were observed for several other microbial taxa, suggesting that the effects of the gut microbiota on transplant outcomes are taxon-specific rather than universal. |
| 15 | **Limitations** | Discuss the limitations of the study, considering the validity of the instrumental variable assumptions, potential sources of bias, and statistical imprecision. Address both the direction and magnitude of any potential biases, and describe the strategies used to mitigate or assess their impact | Article | However, several limitations should be acknowledged. First, transplant rejection is a complex and dynamic process, and alterations in the gut microbiota may occur before, during, or after transplantation as a consequence of immunosuppressive therapy, antibiotic exposure, or perioperative factors, which may complicate causal interpretation. Although Mendelian randomization reduces reverse causation, it cannot fully account for time-varying post-transplant exposures. Second, this study relied on summary-level GWAS data rather than individual-level data, which precluded stratified analyses and the evaluation of specific genetic variants at the individual level. Third, the study population was restricted to individuals of European ancestry, and differences in genetic architecture, gut microbial composition, and transplant practices across populations may limit the generalizability of our findings to other ethnic groups. Finally, for some microbial taxa, the number of available genetic instruments was relatively small (fewer than 10 SNPs), raising the possibility of weak instrument bias and reducing the precision and robustness of the causal estimates. |
| 16 | **Interpretation** |  |  |  |
|  | a) | Meaning: Provide a cautious overall interpretation of the findings, taking into account the study limitations and placing the results in the context of existing evidence and related studies. | Article | Looking ahead, with a deeper understanding of AD and rapid advancements in neuroscience, epidemiology, and related fields, future studies are likely to delve further into the correlation between entertainment activities and AD. Given that MR primarily assesses associated impacts, additional high-quality studies are imperative to investigate the potential benefits of entertainment activities on AD. This research promises to deepen our understanding of AD pathogenesis and provide robust scientific evidence to inform prevention and treatment strategies for the disease. |
|  | b) | Mechanism: Provide a balanced and cautious interpretation of the findings, considering the study’s limitations and situating the results within the context of existing evidence and related literature | Article | Looking ahead, with an improved understanding of transplant immunobiology and rapid advances in microbiome research, genomics, and causal inference methods, future studies are expected to further elucidate the role of the gut microbiota in transplant rejection. Although Mendelian randomization provides valuable evidence supporting potential causal relationships, it primarily captures genetically mediated effects and cannot fully account for dynamic post-transplant factors such as immunosuppressive regimens and antibiotic exposure. Therefore, additional high-quality experimental and clinical studies are warranted to validate these findings and to clarify the mechanistic pathways through which the gut microbiota influences alloimmune responses. Such efforts will deepen our understanding of transplant rejection pathogenesis and facilitate the development of microbiota-targeted strategies to improve long-term transplant outcomes. |
|  | c) | Clinical relevance: Discuss the clinical and/or public health relevance of the findings, and evaluate the extent to which the results inform the magnitude of effects associated with potential interventions. | Article | Further validation of the gut microbiota as a modifiable determinant of transplant rejection could support the development of microbiota-targeted strategies in transplant recipients, offering actionable opportunities to improve graft outcomes and long-term transplant success. |
| 17 | **Generalizability** | Discuss the generalizability of the study findings with respect to (a) other populations, (b) different exposure periods or timings, and (c) varying levels of exposure. | Article | Looking ahead, with a deeper understanding of transplant immunology and rapid advances in microbiome research, genomics, and epidemiology, future studies are expected to further elucidate the role of the gut microbiota in transplant rejection. Although Mendelian randomization provides valuable evidence supporting potential causal relationships, it primarily captures genetically mediated effects and cannot fully account for dynamic post-transplant factors. Therefore, additional high-quality experimental and clinical studies are warranted to validate these findings and to clarify the mechanisms by which the gut microbiota influences alloimmune responses. Such efforts will deepen our understanding of transplant rejection pathogenesis and provide robust scientific evidence to inform the development of microbiota-targeted preventive and therapeutic strategies in organ transplantation. |
|  | **OTHER INFORMATION** |  |  |  |
| 18 | **Funding** | Describe sources of funding and the role of funders in the present study and, if applicable, sources of funding for the databases and original study or studies on which the present study is based | Article | This work was supported by the National Natural Science Foundation of China (82502148), the Department of Science and Technology of Sichuan Province (2025ZNSFSC1743) and The Medical Research Project of Chengdu Municipal Health Commission (2024147). |
| 19 | **Data and data sharing** | Provide the data used to perform all analyses, or clearly state where and how the data can be accessed, and cite these sources within the article. In addition, provide the statistical code required to reproduce the results, or indicate whether the code is publicly available and, if so, specify where it can be accessed. | Article | 2.1-2.2 Study design and data sources |
| 20 | **Conflicts of Interest** | All authors should declare all potential conflicts of interest | Article | The authors declare that the research was conducted in the absence of any commercial or financial relationships that could be construed as potential conflicts of interest. |

This checklist is copyrighted by the Equator Network under the Creative Commons Attribution 3.0 Unported (CC BY 3.0) license.

1.	Skrivankova VW, Richmond RC, Woolf BAR, Yarmolinsky J, Davies NM, Swanson SA, et al. Strengthening the Reporting of Observational Studies in Epidemiology using Mendelian Randomization (STROBE-MR) Statement. JAMA. 2021;under review.

2.	Skrivankova VW, Richmond RC, Woolf BAR, Davies NM, Swanson SA, VanderWeele TJ, et al. Strengthening the Reporting of Observational Studies in Epidemiology using Mendelian Randomisation (STROBE-MR): Explanation and Elaboration. BMJ. 2021;375:n2233.
