# Supplementary material for: Metronidazole-mediated gut anaerobe remodeling is associated with transplant rejection
Source: Front Cell Infect Microbiol. 2026 Aug 18;16:1874387. doi: 10.3389/fcimb.2026.1874387 (PMC13529449; doi:10.3389/fcimb.2026.1874387)
Supplement: Supplementary file 3 [file Table7.docx]

|  | MR method | No. of SNP | OR | 95%CI | *P*-value | *P-*pvalue* |
| --- | --- | --- | --- | --- | --- | --- |
| Intestinibactergenus | IVW | 14 | 0.20 | 0.07-0.614 | 0.004 | 0.45 |
|  | Weighted median | 14 | 0.22 | 0.05-0.93 | 0.040 |  |
|  | Weighted mode | 14 | 0.61 | 0.05-7.67 | 0.708 |  |
|  | MR Egger | 14 | 0.74 | 0.02-22.5 | 0.866 |  |
| Bilophilagenus | IVW | 13 | 0.19 | 0.05-0.65 | 0.008 | 0.26 |
|  | Weighted median | 13 | 0.17 | 0.03-0.91 | 0.038 |  |
|  | Weighted mode | 13 | 0.09 | 0.01-1.54 | 0.122 |  |
|  | MR Egger | 13 | 0.005 | 1.16E-05-2.30 | 0.119 |  |
| Eubacterium fissicatena | IVW | 9 | 2.55 | 1.12-5.78 | 0.03 | 0.21 |
|  | Weighted median | 9 | 2.83 | 0.99-8.05 | 0.0517 |  |
|  | Weighted mode | 9 | 2.41 | 0.51-11.31 | 0.297 |  |
|  | MR Egger | 9 | 0.14 | 0.002-10.09 | 0.401 |  |
| Ruminococcus | IVW | 15 | 0.32 | 0.11-0.91 | 0.033 | 0.57 |
|  | Weighted median | 15 | 0.30 | 0.06-1.38 | 0.122 |  |
|  | Weighted mode | 15 | 0.33 | 0.04-2.78 | 0.323 |  |
|  | MR Egger | 15 | 0.32 | 0.02-4.26 | 0.404 |  |
| Defluviitaleaceae | IVW | 9 | 0.30 | 0.09-0.99 | 0.048 | 0.9 |
|  | Weighted median | 9 | 0.40 | 0.08-1.98 | 0.263 |  |
|  | Weighted mode | 9 | 0.47 | 0.05-4.67 | 0.538 |  |
|  | MR Egger | 9 | 0.39 | 0.005-27.59 | 0.676 |  |

*P-pval, horizontal pleiotropy p value.
